# Mutations in a novel serine protease *PRSS56* in families with nanophthalmos

**Published:** 2011-07-12

**Authors:** Andrew Orr, Marie-Pierre Dubé, Juan C. Zenteno, Haiyan Jiang, Geraldine Asselin, Susan C. Evans, Aurore Caqueret, Hesham Lakosha, Louis Letourneau, Julien Marcadier, Makoto Matsuoka, Christine Macgillivray, Mathew Nightingale, Simon Papillon-Cavanagh, Scott Perry, Sylvie Provost, Mark Ludman, Duane L. Guernsey, Mark E. Samuels

**Affiliations:** 1 Department of Ophthalmology and Visual Sciences, Dalhousie University, Halifax, Nova Scotia, Canada; 2 Montreal Heart Institute, Montreal, Quebec, Canada; 3 Department of Biochemistry, Faculty of Medicine, National Autonomous University of Mexico (UNAM), and Department of Genetics-Research Unit, Institute of Ophthalmology "Conde de Valenciana", Mexico City, Mexico; 4 Department of Pathology, Dalhousie University, Halifax, Nova Scotia, Canada; 5 Centre de Recherche du CHU Ste-Justine, Université de Montréal, Montreal, Quebec, Canada; 6 McGill University and Génome Québec Innovation Centre, Montréal, Quebec, Canada; 7 Department of Pediatrics, Division of Medical Genetics, IWK Health Centre and Dalhousie University, Halifax, Nova Scotia, Canada; 8 Maritime Medical Genetics Service, IWK Health Centre, Halifax, Nova Scotia

## Abstract

**Purpose:**

Nanophthalmos is a rare genetic ocular disorder in which the eyes of affected individuals are abnormally small. Patients suffer from severe hyperopia as a result of their markedly reduced axial lengths, but otherwise are capable of seeing well unlike other more general forms of microphthalmia. To date one gene for nanophthalmos has been identified, encoding the membrane-type frizzled related protein MFRP. Identification of additional genes for nanophthalmos will improve our understanding of normal developmental regulation of eye growth.

**Methods:**

We ascertained a cohort of families from eastern Canada and Mexico with familial nanophthalmos. We performed high density microsatellite and high density single nucleotide polymorphism (SNP) genotyping to identify potential chromosomal regions of linkage. We sequenced coding regions of genes in the linked interval by traditional PCR-based Sanger capillary electrophoresis methods. We cloned and sequenced a novel cDNA from a putative causal gene to verify gene structure.

**Results:**

We identified a linked locus on chromosome 2q37 with a peak logarithm (base 10) of odds (LOD) score of 4.7. Sequencing of coding exons of all genes in the region identified multiple segregating variants in one gene, recently annotated as serine protease gene (*PRSS56*), coding for a predicted trypsin serine protease-like protein. One of our families was homozygous for a predicted pathogenic missense mutation, one family was compound heterozygous for two predicted pathogenic missense mutations, and one family was compound heterozygous for a predicted pathogenic missense mutation plus a frameshift leading to obligatory truncation of the predicted protein. The *PRSS56* gene structure in public databases is based on a virtual transcript assembled from overlapping incomplete cDNA clones; we have now validated the structure of a full-length transcript from embryonic mouse brain RNA.

**Conclusions:**

*PRSS56* is a good candidate for the causal gene for nanophthalmos in our families.

## Introduction

Nanophthalmos [OMIM 600165] is characterized by a very small but structurally intact and functional eye, leading to extreme farsightedness or hyperopia. As such, it is the extreme anti-phenotype of common myopia (nearsightedness). It thus offers tantalizing insights into emmetropization, the exacting process by which the eye’s postnatal growth is regulated in such a way to deliver a focused image to the retina. The homeostatic mechanisms governing such tightly regulated growth remain mysterious, although they appear to involve feedback guided by visual stimuli [1,2].

There are several genetic disorders and identified genes in which the eyes are small and disorganized to varying extents, a condition defined generically as microphthalmia and in the extreme anophthalmia if the eyes are almost completely absent. These are thought to result generally from defects in genes encoding transcription factors critical to the eye’s early formation. Nanophthalmic patients are in principle distinct as the eye is otherwise well-formed and fully functional. Although they may suffer problems associated with the extreme small size (such as increased risk of glaucoma, uveal effusion and the potential for amblyopic vision loss), these seem secondary to the actual disease state, which may be viewed as one extreme on the spectrum of refractive errors [3].

To date one gene has been identified for isolated nanophthalmos, the membrane-type frizzled-related protein (*MFRP*) [OMIM 606227] associated with genetic locus NNO2 [OMIM 609549] on chromosome 11q23.3 [4,5]. Other mutations in *MFRP* have however been found in patients with additional retinal defects [6-9], as well as in a mouse model with retinal degeneration [10], thus the involvement of this gene in the regulation of eye size per se remains to be clarified. A dominant form of nanophthalmos, NNO1 [OMIM 600165] has been linked to chromosome 11 in a large region including the centromere, but no causal gene has been reported [11]. Another form of the disease has been linked to a 16 Mb region on chromosome 2q11-14 in a large Chinese family as locus NNO3 [OMIM 611897], although the phenotype is described as simple microphthalmia rather than nanophthalmos, and no gene has been reported [12]. One allele of the gene bestrophin 1 (*BEST1*), [OMIM 607854], is reported to cause dominant vitreoretinochoroidopathy together with nanophthalmos, although the relationship of this phenotype to pure nanophthalmos seems uncertain [13]. There is some disagreement in the field as to the precise differential definitions of the various forms of microphthalmia and nanophthalmos [14].

We ascertained several families with a pure, non-syndromic form of nanophthalmos segregating as an apparent autosomal recessive genetic disorder. Through marker-assisted linkage mapping, we mapped a novel chromosomal locus. By direct DNA sequencing, we identified a gene in the region segregating multiple different potentially pathogenic variants in the affected individuals. The gene, *PRSS56,* previously annotated only as an anonymous transcript LOC646960, is a strong candidate for the causal gene in our families.

## Methods

Approval for this study was obtained from the Research Ethics Board of the Queen Elizabeth II Health Sciences Centre, Halifax, Nova Scotia, Canada.

### Clinical ascertainment and consent

Patients were identified in the course of clinical practice of two of us (A.C.O., J.C.Z.). All sampled family members provided informed consent to participate in the study. DNA was obtained from blood samples using routine extraction methods. All procedures were in accordance with ethical and methodological standards for human experimentation.

### Genotyping and analysis

Whole genome high density single nucleotide polymorphism (SNP) genotyping scanning was performed at the McGill University and Genome Quebec Centre for Innovation, using the Illumina HumanHap300 (Illumina, Inc., San Diego, CA) panel (some individuals were genotyped with the 300K v1 [317,503 SNPs] and others with the 300K v2 [318,237 SNPs]). There are 311 398 SNPs in common between the two assays. Analyses were done on those SNPs shared in common between the two versions. Data were scanned using the Bead Array Reader (Illumina, Inc.), plate Crane Ex, and Illumina BeadLab software (Illumina, Inc.). Preliminary SNP scans were performed using the Affymetrix Xba240 and Hind240 (Affymetrix, Inc. Santa Clara, CA) panels including approximately 50,000 markers each. Microsatellite genotyping was performed genome-wide by deCODE Genetics, Inc. (Reykjavik, Iceland), or locally.

### Linkage analysis

Genotyped data were imported into a Microsoft Access Database (Microsoft Corporation, Redmond, WA) used in previous analysis. Queries were used to produce the genotype and pedfile.pro files in a format compatible with ALOHOMORA [15].

Data were imported into ALOHOMORA_M (Mega-Chips) v0.33.0 a software tool designed to convert SNP data into appropriate format for MERLIN linkage analysis software [16]. Genders were checked with data quality tools from ALOHOMORA_M. GRR was used to evaluate familial relationship. Mendelian inconsistencies were detected with the ALOHOMORA interface using Pedcheck v1.1 [17]. All genotypes with Mendelian errors were deleted with the option *delete ME.* The *–error* option from MERLIN was also used to detect unlikely genotypes. These genotypes were zeroed out before analysis. Genotype data from 60 unrelated individuals (CEU) were downloaded from the Illumina ftp site in order to calculate allele frequencies with the SAS genetic v9.1.3 *proc allele* procedure.

The genetic map provided by Illumina contained some discrepancies between the genetic and the physical position. A distance of 0.001 cM was added when two or more markers have exactly the same genetic position. Order was based on the physical position. Physical positions were taken from HumanHap300_v2.0_Annotation.zip downloaded from the Illumina ftp site.

The analyses were conducted with parameters previously used: a penetrance set to 0.95, a phenocopy rate of 0.001 for a recessive disease with an allele frequency of 0.01.

The protocol “How to prepare files for Alohomora with a selection of tagSNPs” was used to select tag SNPs for multipoint and haplotyping analysis as follows:

The SNPs were first selected with Haploview version 4.1 with an r^2^ of 0.18 then SNPs with an A allele frequency <0.2 were removed and a selection based on genetic position was applied, SNPs spaced by 0.1 cM were kept. Selection by frequency and genetic position was done with tagSNP_selector.py created by Louis-Philippe Lemieux Perreault, Statistical Genetics, Montreal Heart Institute, University of Montreal, Montreal, Quebec, Canada. A total of 17 355 SNPs were selected for the analysis.

Protocol “MERLIN from Alohomora (v0.33.0) for a genome wide analysis” was used for multipoint linkage analysis. Multipoint linkage study was carried out using MERLIN version 1.1.2 on Linux. Script merlin_start_multi_pl.pl created by Geraldine Asselin was used to launch the analysis. The LOD scores were compiled by extracting results from the Merlin output files with compile-merlin.pl by Geraldine Asselin. Graphical representation was done on Linux with the create_linkage_graph.py application (version 7).

Option *--best* of Merlin version 1.1.2 was used for haplotype reconstruction. Haplotypes were compiled with compile-haplo-merlin.pl script created by Geraldine Asselin. An Excel (Microsoft Corporate Headquarters) macro was used to color the different haplotype in Excel workbooks.

### Mutation detection and analysis

Annotated coding exons were amplified by PCR using standard methods, and sequenced at the McGill University and Genome Quebec Centre for Innovation, and Dalhousie University, using Sanger fluorescent sequencing and capillary electrophoresis. Sequence traces were analyzed using MutationSurveyor (Soft Genetics LLC. State College, PA) Specific primers for amplification of *PRSS56* exons and PCR conditions are provided in Table 1.

**Table 1:** PCR primers for sequencing *LOC646960*.

**Sequence 5'→3'**	**Primer name**
attcccctgtgggctccta	LOC646960_E01_F
gtccttatgagtgggggtga	LOC646960_E01_R
gctcacttgcctcctcattc	LOC646960_E02_F
tccactcggagagacagacc	LOC646960_E02_R
gaaaggagagatggggagaga	LOC646960_E03E04_F
ggcagcagagaccaccttt	LOC646960_E03E04_R
gcccccaggtggagaaag	LOC646960_E05E06_F
aagagcaggcagcatttttc	LOC646960_E05E06_R
tctttcaaagggggaggaat	LOC646960_E07E08_F
ggtcagctcaccctctgttt	LOC646960_E07E08_R
cgggaaagcctgtctcct	LOC646960_E09E10_F
tcattaccgttggcttctcc	LOC646960_E09E10_R
ctgcggcttcactcaggta	LOC646960_E11_F
ccatggggtaagcccttt	LOC646960_E11_R
gcctcagtttccccacctat	LOC646960_E12_F
ctcggaccctctacctaccc	LOC646960_E12_R
gaaatgagcagggtttccag	LOC646960_E13_F
ttgtaaacctgggaagacacg	LOC646960_E13_R
gaatgcagcgtcctctctct	LOC646960_V302F_F
agccagtccttgaacactgc	LOC646960_V302F_R

### Array capture and next-generation sequencing

For array-capture next-generation sequencing, a custom tiling array was designed in collaboration with Roche Nimblegen, Inc. (Madison, WI), to cover a region slightly larger than the entire linked interval on chromosome 2 (4 million base pairs [Mb], from 229,900,000 bp to 233,900,000 bp in genome assembly hg18). Probes were constrained to unique sequence within the segment, thus there were internal gaps corresponding to the positions of repetitive elements. Samples from affected patients 172 (family 1) and 1376 (family 2) were captured at Roche Nimblegen, Inc., and submitted for Roche/454 Titanium sequencing at the McGill University and Genome Quebec Centre for Innovation. Mean read depth for on-target bases was 2×, and the mode of read length was 500 nt (with the mean length slightly shorter). Data were analyzed using NextGene from SoftGenetics, Inc., and separately using software developed at the Broad Institute.

### Bioinformatic analysis

The functional significance of putative pathogenic missense variants in *PRSS56* was analyzed using PolyPhen2 [18], which generates its own set of homologous sequences from database searches. In this case we noted that the PolyPhen2 dataset included genes unlikely to be true orthologs. Therefore, we also used the human *PRSS56* reference sequence (NP_001182058) in a BLAST search to identify potential orthologs in other species. High-scoring BLASTP hits were individually reviewed for chromosomal synteny based on neighboring genes in the human reference assembly (*ECEL1* [endothelin-converting enzyme-like 1] and *CHRND* [cholinergic receptor, nicotinic, delta] to left and right respectively); likely orthologs were identified in dog (*C. familiaris*, XP_852751.1), chicken (*G. Gallus*, XP_422746.2), zebra finch (*T. guttata*, XP_002191450.1), macaque (*M. mulatta*, XP_001109183.2), orangutan (*P. abelii*, XP_002813037.1), marmoset (*C. jacchus*, XP_002749985.1), European rabbit (*O. cuniculus*, XP_002721490.1), mouse (*M. musculus*, XP_911207.4), rat (*R. norvegicus*, D3ZQJ8), green spotted pufferfish (*T. nigroviridis*, Q4RV82), lancelet (*B. floridae*, C3Y046). The chimpanzee (*P. paniscus*) ortholog was excluded as its identity to the human is too high to be useful in variant functional predictions. All sequences were aligned using MUSCLE [19] or MAFFT [20] with similar results, and displayed with BOXSHADE. An independent set of approximately 90 functionally annotated proteins containing Tryp-SPc protease domains was also obtained for CONSURF analysis. Analyses of putative causal mutations was performed using Phyre [21], SIFT [22], and CONSURF [23]. For identification of predicted functional activities, the *PRSS56* human sequence (NP_001182058) was used to query the NCBI CD-Search database.

### Molecular cloning

For cloning a cDNA for *PRSS56*, we used oligo-dT primed first strand cDNA of mouse embryonic brain stage E12.5 with Superscript II (Life Technologies Invitrogen, Carlsbad, CA). The predicted full length open reading frame was amplified using forward primer 5'-GCA AGC TTA CCA TGC CGC TGG CTA TGT T-3' (including a HindIII restriction site for shuttling) and reverse primer 5'-GCG AAT TCT CAC AGG GTT GCC TGG TTC A-3' (including an EcoRI site for shuttling). PCR was done with KOD Hot Start polymerase (EMD Chemicals USA, Gibbstown NJ), with the following conditions: 2 min at 95 °C, 30× (20 s at 95 °C, 10 s at 56 °C, 30 s at 68 °C). Sequence of the cDNA clone was verified using primers in vector sequence plus gene-specific primers based on the predicted exon structure.

## Results

### Clinical assessment and phenotyping

**Figure 1:**
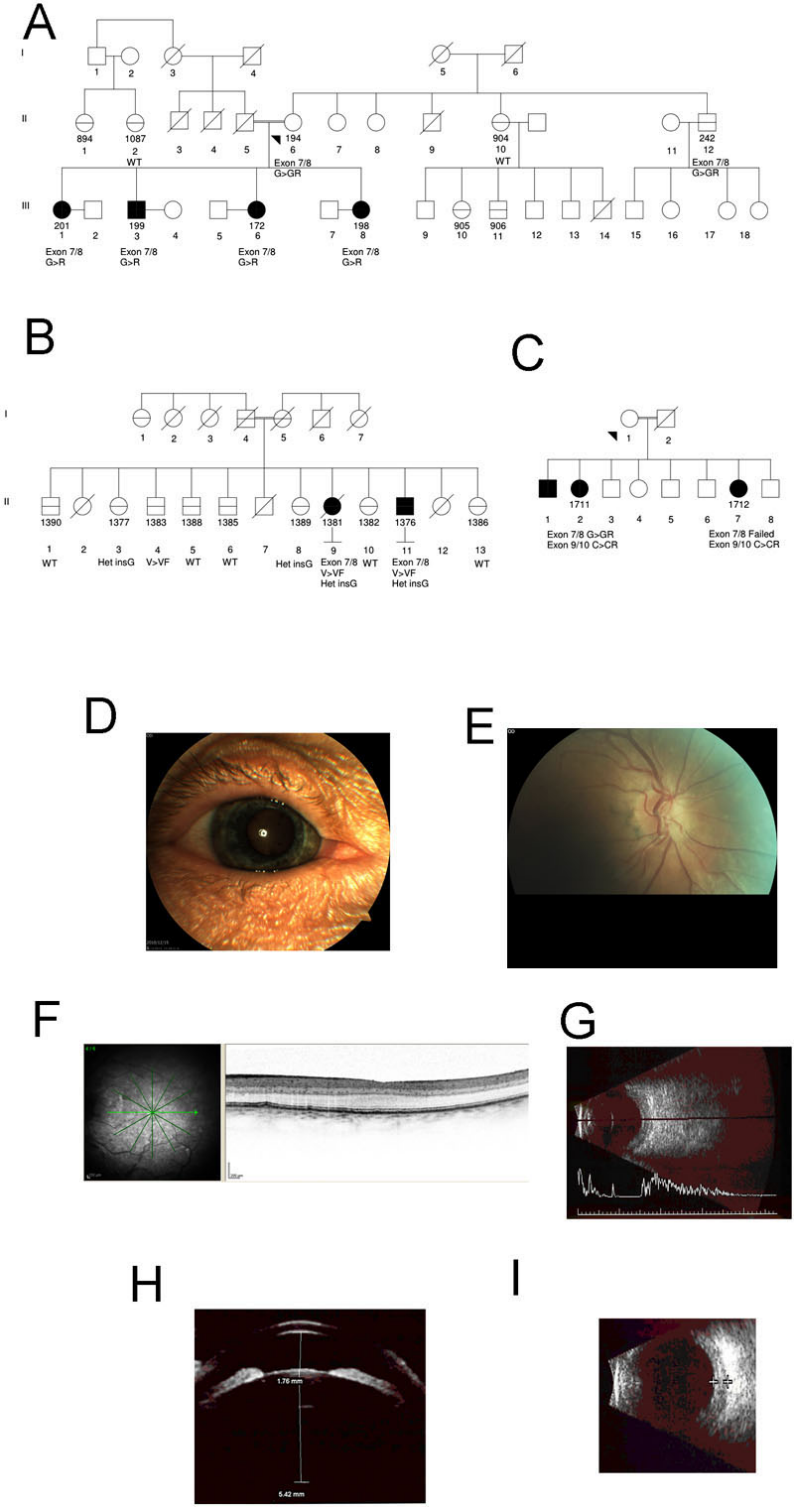
Nanophthalmos families. In each panel, affected individuals are shown with filled black symbols. Sampled individuals have additional identification number in addition to generation numbers. For sampled individuals, genotypes are shown for relevant putative coding mutations in *PRSS56*. **A**: Family 1, (Maritime, mutation p.G320R). Consanguinity results from a closed inheritance loop higher in the pedigree, not shown. **B**: Family 2 (Maritime, mutations p V302F, c.828_833 het_ insG). **C**: Family 3 (Mexico, mutations p.G237R, p.C395P). **D**-**I**: Clinical imaging of the right eye of patient 172: **D**: external photo, with rigid contact lens in situ; **E**: color disc image; **F**: optical coherence tomography of the macula; **G**: contact B-scan ultrasound; **H**: ultrasound biomicroscopy, with anterior chamber and lens thickness dimensions; **I**: choroidal thickening on Immersion B-scan ultrasound.

In the course of routine clinical practice one of us (A.C.O.) ascertained two families from an eastern Canadian Maritime province with multiple affected individuals suffering from nanophthalmos (Figure 1A,B). In both families, only children were affected, consistent with a recessive genetic mode of inheritance. Both families are of anglophonic ethnicity, not otherwise specified, from a region known to descend from early settlement by English, Scottish, and Irish founders.

The first family (F1) consists of an unaffected mother and four affected adult offspring (three females and one male), all of whom have a pure, severe nanophthalmic phenotype. The father, who is dead, did not wear glasses until his presbyopic years and is therefore very unlikely to have been affected. The index case presented with a spontaneous hyphema of unknown etiology, which resolved uneventfully. The outstanding clinical feature of the eyes of the four affected individuals was their remarkably small size, and correspondingly severe hyperopia. Axial lengths ranged from 15.32 to 16.12 mm with a mean of 15.71 mm. (adult normal mean 23.6 mm, standard deviation 0.7 mm) [24] (Alcon Biophysic OcuScan, Clermont, France), and the mean spherical equivalent of refractive errors between +13.25 and +17.00 diopters (D; mean, 15.00 D). Two of the four siblings had amblyopic loss of vision in one eye, with best corrected acuities in the poorer eye ranging between 20/80 and 20/160; the remaining two siblings had mild bilateral depression of visual acuity to between 20/25 and 20/40. Color vision by AO-HRR testing was measured in one sibling (172) and found to be normal. The central corneal thickness (CCT) in the same individual was rather low, measuring 481 and 496 microns in the right and left eyes, respectively (IOPac Standard, Reichart GmbH, Seefeld, Germany). Anterior chambers appeared shallow in all siblings and angles were rated as narrow, but not occludable. The crystalline lenses were large, displacing the iris anteriorly. Thus far there have been no instances of angle-closure glaucoma, and the intraocular pressures have remained within the normal range without treatment. Three of the four siblings underwent dilated examinations under pharmaceutical mydriasis, carried out uneventfully without evidence of angle narrowing or elevation of intraocular pressure (IOP). In all cases the optic nerves were small and rather congested and lacked a discernable cup. Morphologically, the maculae appeared to be grossly normal although no foveal reflex was seen. The peripheral retina was flat to the ora serrata. All of the siblings were systemically well.

Electrodiagnostic testing and detailed imaging was carried out in one member (patient 172) of this pedigree. This revealed normal visual evoked potentials (VEPs), and nonspecific reduction in the amplitude of electroretinography (ERG) potentials, possibly related to their being carried out in an undilated state. Contact and immersion B-scan ultrasonography revealed a small eye with a crystalline lens appeared normally positioned, but large in size relative to that of the globe. The choroid was diffusely thickened in all four quadrants, measuring 2.3 mm at the posterior pole. Ultrasound biomicroscopy (UBM) confirmed these observations, documenting an average anterior chamber depth of 1.62 mm and an average anteroposterior (AP) lens thickness of 4.91 mm (representing approximately one third of the total AP extent of the eye). Optical coherence tomography (Spectralis OCT; Heidelberg Engineering, Heidelberg, Germany) of the macula revealed a grossly normal structure.lacking a foveal depression, consistent with previous reports of macular hypoplasia (Figure 1 D-I) [25].

The second family (F2) consists of two affected and eight unaffected offspring of two unaffected parents. The clinical features of this family were similar to that of the first, except that the axial length was somewhat greater (mean, 17.59 mm), hyperopia somewhat less (mean spherical equivalent, 12.48 D) and glaucoma was present in both affected individuals. According to the medical records, one affected subject (1381) presented in angle closure glaucoma with visual acuities of no light perception and hand motions, and treated IOPs of 36 and 50 mm of mercury in the right and left eyes, respectively. Ultimately she went on to surgical peripheral iridectomy in the left eye, which was complicated by a severe uveal effusion. The other affected sibling (1376) maintained relatively good vision (20/70 and 20/60, respectively) and medically controlled intraocular pressures without any episodes of angle closure. Like subject 172 from the first family, his central corneas were rather thin (measuring 457 and 459 microns, respectively) His optic nerves appeared small and congested; the posterior pole was morphologically within normal limits. He eventually underwent uncomplicated phacoemulsification surgery in the right eye with placement of a 40 diopter SA60AT intraocular lens (Alcon Labs, Ft. Worth, TX).

A third family (F3), ascertained in Guerrero State, Mexico by one of us (J.C.Z.), consists of three affected and five unaffected siblings (Figure 1C). The mean axial length of the two examined affected members was 16.43 microns and mean spherical equivalent refractive error was 19.5. Visual acuities ranged from 20/200 to 1/200 The index patient presented with features of chronic angle closure, retinal vascular tortuosity, asteroid hayalosis, a small optic nerve and absence of a macular light reflex and choroidal thickening noted on A-scan ultrasound; the other sibling was found to have a shallow anterior chamber but declined further examination.

The mother and father in family 1 are second cousins. Seven relatives were available for examination and were also determined to be unaffected, although a male first cousin was reported to have a cone-rod dystrophy. Overt consanguinity was not detected in family 2, although the same surname was shared by four of eight great-grandparents. Notwithstanding the similarity of the phenotype and the relative (80 km) proximity of families 1 and 2, we were unable to find any evidence of a genealogical connection between them. Consanguinity was also absent from the third family. No obvious phenotypic carrier state has been discovered in any of the relatives of affected patients in the three families.

### Molecular genetic analysis

**Figure 2:**
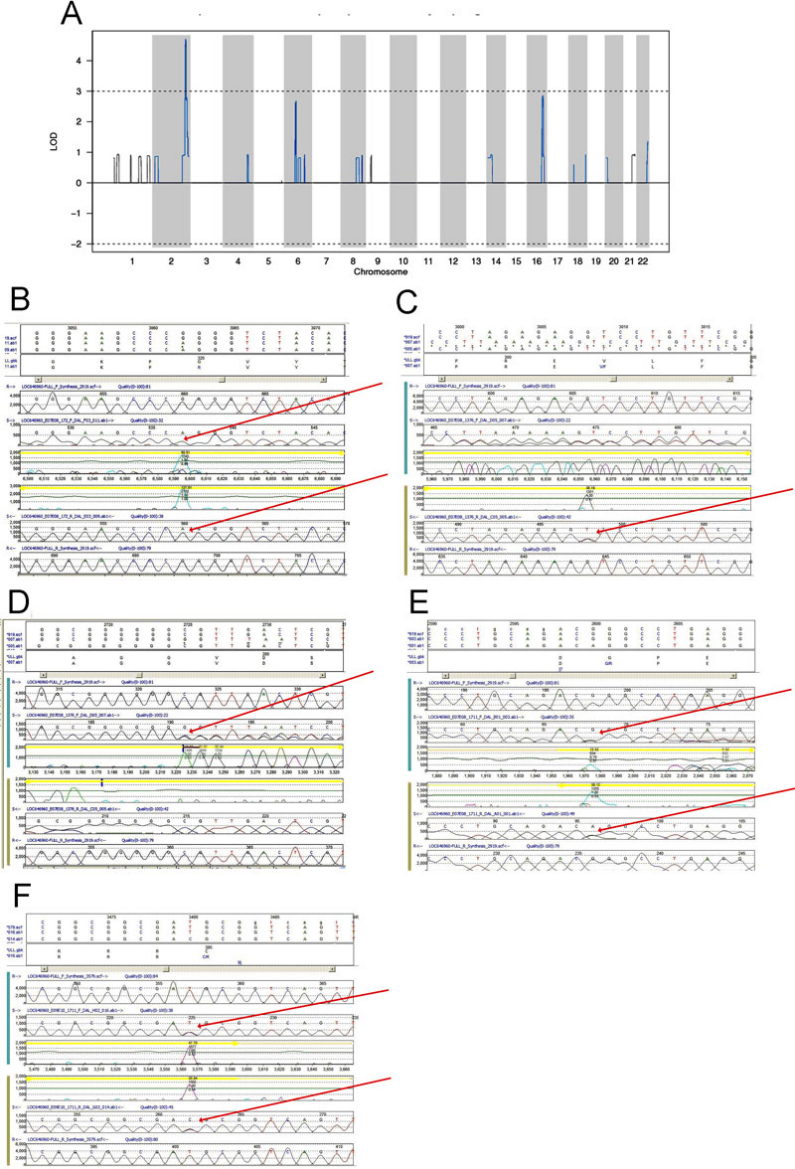
Linkage and mutation analysis. **A**: Multipoint heterogeneity LOD score for families. **B**-**F**: Sequence chromatograms for putative causal mutations in *PRSS56* in families 1, 2 and 3. In each panel, upper to lower tracks contain translation of coding exon in consensus and mutated sequences; virtual chromatogram of consensus genomic sequence forward direction (generated by software from text sequence); sequence chromatogram of affected patient reverse direction; virtual chromatogram of consensus genomic sequence reverse direction. Red arrows point to mutations in patient samples. **B**: p.G320R homozygous in affected patient from family 1. **C**: p V302F heterozygous in affected patient from family 2. **D**: c.828_833 het_ insG heterozygous in affected patient from family 2. **E**: p.G237R heterozygous in affected patient from family 3. **F**: p.C395P heterozygous in affected patient from family 3.

The absence of phenotype in the parents of all affecteds, and the pattern of affection, were consistent with an autosomal recessive disorder. Consanguinity in family 1 further suggested the likelihood of homozygosity at least in that family. In preliminary work we performed a whole genome scan on family 1 using a set of microsatellite markers. Homozygosity mapping was suggestive of linkage to a region on chromosome 2q (data not shown) Subsequently we subjected all sampled individuals from families 1 and 2 to whole genome genotyping with single nucleotide polymorphisms (SNPs), initially with 100,000 and ultimately with a high density panel of 311,400 SNP markers. Formal linkage analysis identified the same region on chromosome 2q37 as originally detected with the microsatellite genome scan but with significantly greater resolution (Figure 2A). The two families together generated a combined hetLOD score of 4.7, achieving genome-wide statistical significance. By phased haplotype analysis, all four affected children in family 1 were homozygous for a shared haplotype in the linked region. The two affected children in family 2 were compound heterozygous for two parental haplotypes, both different than the homozygous linked haplotype in family 1, and all 8 unaffected siblings carried other haplotype combinations (data not shown). Although linkage in both families formally excluded the known nanophthalmos causal gene *MFRP*, we sequenced the protein-coding regions of that gene as well as *BEST1* in one patient from each family, but detected no interesting variants in either gene (data not shown). No pathogenic variants were found in the third family by sequencing *MFRP* in one affected patient.

The linked region, from marker rs1477111 to rs7563345, covers 3,681,314 bp in the current human genome assembly hg19, and includes 46 annotated protein-coding genes, 1 miRNA and 3 small nuclear RNAs. We sequenced all annotated coding exons for these genes and RNAs by PCR-based Sanger capillary sequencing (a total of 463 exons). At first no variants were identified which could explain the disorder in the two genotyped families. However, following ongoing revisions to the gene annotation in this region with continued mutation detection, potentially pathogenic variants were eventually identified in a newly annotated gene, *PRSS56*, previously identified as anonymous transcript LOC646960 in the RefSeq NCBI database. In family 1, a missense variant, p.G320R was observed (Figure 2B). The variant was homozygous in all four affected children, and heterozygous or wild-type in the 6 other sampled unaffected family members (heterozygous in the one sampled parent; Figure 1A). The variant is not present in dbSNP v.131, nor was it observed in 246 sequenced controls including 96 CEPH samples plus 150 local controls of anglophonic or francophonic ethnicity. In family 2, two variants were detected by exon sequencing: missense p.V302F, and c.828_833 het_ insG which creates a framshift and subsequent premature termination codon yielding a 279 amino acid protein compared to the wild-type 603 amino acid full-length predicted protein. The two affected children were both compound heterozygous for the two variants, and all unaffected siblings were singly heterozygous for one or the other variant consistent with the phased haplotype analysis (parental samples were not available; Figure 1B and Figure 2C,D). The frameshift is not present in dbSNP v.131 nor in the 300 local control chromosomes. The missense variant p.V302F is present in dbSNP v.131 as rs74703359, reported as heterozygous in one CEU sample from the 1000 Genomes pilot project. However it was not observed in our 96 CEPH plus 150 local control samples and could potentially represent a CEU sample cell line artifact. The human wild type consensus sequence allele (G) is the ancestral allele in other primate genomes (chimpanzee, orangutan, macaque) at the orthologous position.

In a separate experiment, we subjected the entire linked chromosomal region to hybrid capture and genome resequencing. We designed a tiled array of oligonucleotide probes covering the entire non-repetitive content of the linked region. DNA from two affected patients, one from each family 1 and 2, was separately hybridized to the array and the captured material was sequenced using Roche/454 Titanium chemistry. Analysis of these results was consistent with the results of PCR-based exon resequencing; no additional coding region variants were detected, and the three variants in *PRSS56* from the two families were confirmed.

In family 3, ascertained after linkage had already been obtained, we sequenced the entire coding region of *PRSS56*. The affected individuals carried two novel missense heterozygous variants, p.G237R and p.C395R (Figure 1C and Figure 2E,F). We were unable to sample other family members to verify that the two variants are in trans in the patients.

A second Mexican family with nanophthalmos had a homozygous c.30A>T of unlikely genetic significance in *PRSS56*. No other novel coding variants were found in the gene in this family, or in the coding region of *MFRP*, suggesting the possibility of additional genetic heterogeneity for this disorder.

### Structure of *PRSS56*

**Figure 3:**
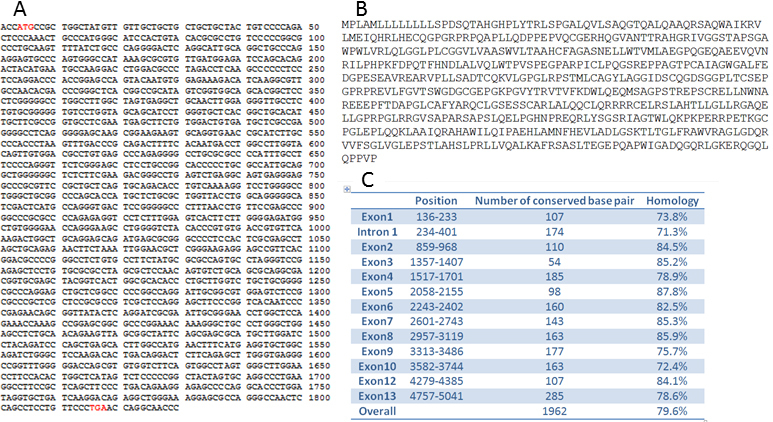
Mouse cDNA for *PRSS56*. **A**: Sequence of cloned mouse cDNA, ortholog of *PRSS56*, from embryonic brain RNA library. Start and stop codons are in red. **B**: Predicted sequence of mouse protein ortholog of PRSS56. **C**: Percent identity of mouse exons from cDNA clone and predicted human exons from annotated database.

*PRSS56* is incompletely annotated in the NCBI database. Its structure was initially defined by several overlapping incomplete cDNA clones, from human and mouse and/or pig. To verify the transcript structure, we amplified the predicted full-length open reading frame using primers from the predicted first and last coding exons, using first strand cDNA generated from mouse embryonic brain RNA. A product of the correct length was obtained based on the predicted exon structure (data not shown). Sequencing of a clone from this product was fully consistent with the predicted exon structure in both mouse and human, and was 100% identical across the mouse consensus genome sequence (Figure 3), and is also consistent with a recently published cDNA clone sequence.

### Bioinformatic analysis of PRSS56

**Figure 4:**
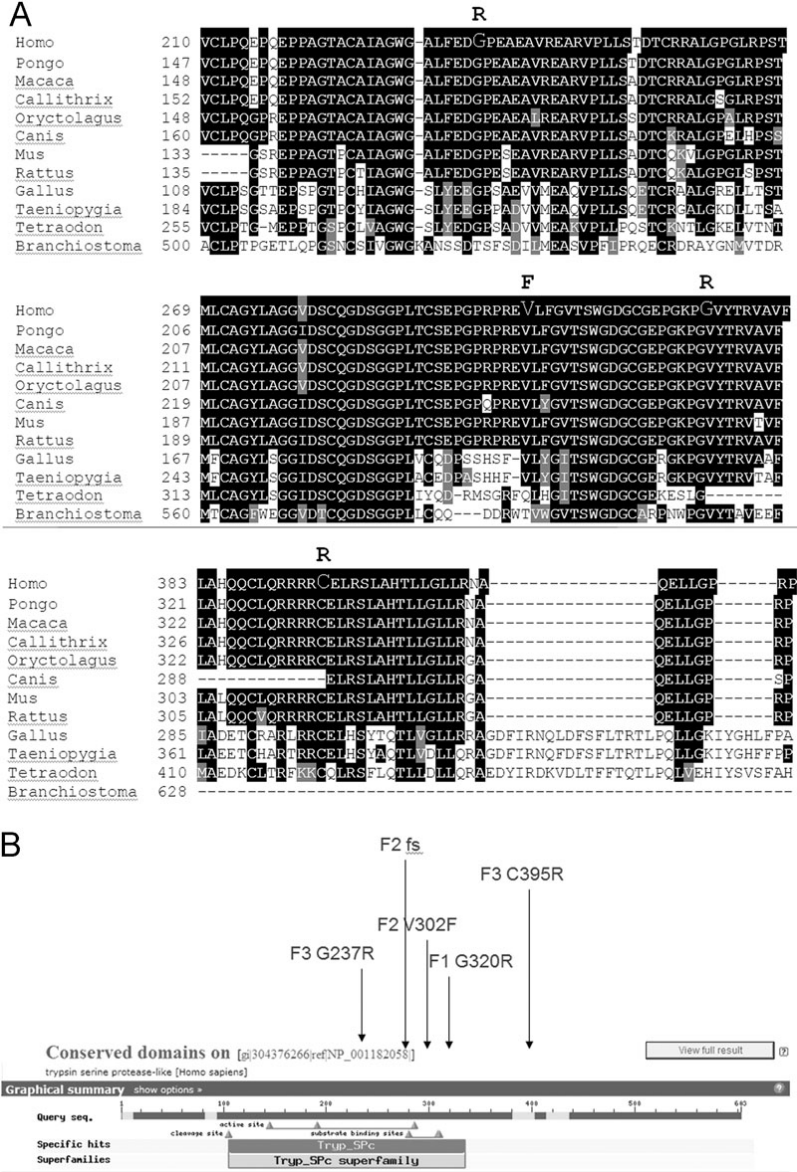
Multiple sequence alignments of *PRSS56*. **A**: Alignment of putative orthologs from multiple species, around locations of four familial missense variants believed to be pathogenic. Human sequence is top row of each subpanel, with mutated residue in larger font, with mutation in bold above human sequence. **B**: Predicted trypsin-like serine protease activity by NCBI Conserved Domains database with positions of mutations observed in our NNO families.

Although there are very few cDNA clones from other species, *PRSS56* has potential putative orthologs in other sequenced vertebrate genomes. At the positions of the four missense mutations found in our nanophthalmos patients, the human residue is typically highly conserved (Figure 4A). From the primary amino acid sequence, PRSS56 is predicted to contain a well-documented serine protease enzymatic domain, with several familial mutations lying in the conserved region (Figure 4B).

**Figure 5:**
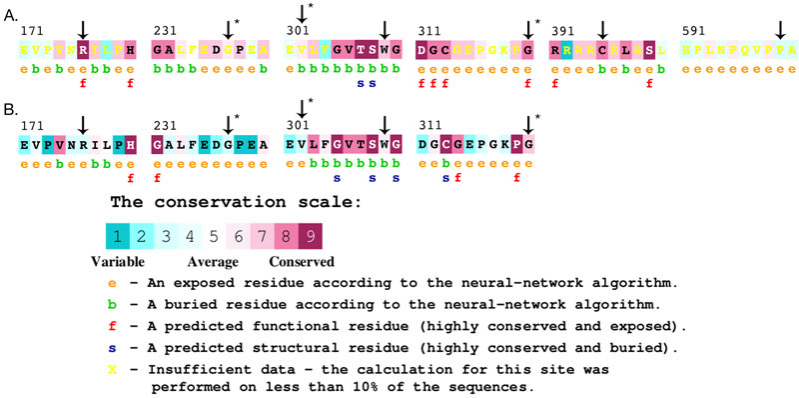
Mutation analysis by CONSURF. **A**: Functional effects of missense mutations observed in our patients (indicated with asterisks, positions 237, 302, 320, and 395) and in published reports (positions 176, 309, and 599), predicted using our set of putative orthologs obtained by BLAST of NCBI genomic databases and syntenic alignment. **B**: Functional effects of missense mutations observed in our patients and in published reports, predicted in comparison to a set of approximately 90 functionally annotated proteins containing Tryp-SPc protease domains. Mutations at positions 395 and 599 are not accessible to comparison using the set of protease domain-containing genes.

PolyPhen2 and SIFT predicted that most of the familial missense variants observed in our study, as well as the two other studies of PRSS56, may be pathogenic (Table 2, Table 3, and Table 4). We employed CONSURF to predict pathogenicity based on either of two basis sets, either a set of likely true orthologs (Figure 5A), or a set of 90 related protease domain-containing proteins (Figure 5B, not all mutations analyzed due to lack of representation in gene set). Several of the putative mutations were less likely to be pathogenic by CONSURF analysis, although among our four missense variants p.G320R and p.C395 were still so predicted. Phyre also predicted that p.G320R is pathogenic.

**Table 2:** PolyPhen2 HumDiv results of familial missense variants in *PRSS56*.

**Mutation**	**Result**	**Score**	**Sensitivity**	**Specificity**
R176G	Probably Damaging	0.981	0.74	0.96
G237R*	Probably Damaging	0.998	0.27	0.99
V302F*	Possibly Damaging	0.917	0.81	0.94
W309S	Probably Damaging	1.000	0.00	1.00
G320R*	Probably Damaging	1.000	0.00	1.00
C395R*	Probably Damaging	0.998	0.27	0.99
P599A	Benign	0.000	1.00	0.00

*Denotes variants observed in the present study.

**Table 3:** PolyPhen2 HumVar results of familial missense variants in *PRSS56*.

**Mutation**	**Result**	**Score**	**Sensitivity**	**Specificity**
R176G	Possibly Damaging	0.522	0.82	0.81
G237R*	Probably Damaging	0.940	0.64	0.92
V302F*	Benign	0.480	0.83	0.80
W309S	Probably Damaging	0.989	0.48	0.96
G320R*	Probably Damaging	0.991	0.45	0.97
C395R*	Probably Damaging	0.923	0.66	0.91
P599A	Benign	0.001	0.99	0.08

*Denotes variants observed in the present study.

**Table 4:** SIFT results of familial missense variants in *PRSS56*, with basis set of putative true orthologs.

**Mutation**	**Result**	**Score**	**Representants**
R176G	Affect protein function	0.00	11
G237R*	Affect protein function	0.01	12
V302F*	Affect protein function	0.01	12
W309S	Affect protein function	0.00	12
G320R*	Affect protein function	0.00	12
C395R*	Affect protein function	0.00	10
P599A	Affect protein function	0.00	4

*Denotes variants observed in the present study.

## Discussion

In two of our families (one Canadian, one Mexican), we identified two novel likely pathogenic mutations in the gene consistent with a recessive genetic mode of inheritance. In the other Canadian family, we identified one novel likely pathogenic truncating mutation, as well as a second missense SNP found in one heterozygote in dbSNP and also potentially pathogenic. Conceivably this family has an additional allelic mutation in a non-coding region (such as in a cryptic intronic splice site or promoter element) in trans to the truncating variant. Alternatively, the missense variant in dbSNP may be pathogenic, and rare enough so that homozygotes are not observed in the general population. dbSNP reports an allele frequence of 1.4% for this variant, but in fact it was only observed once as a heterozygote in the CEU HapMap samples and not at all among the 1000Genomes samples (sequenced to variable coverage), so the true frequency in populations is unknown; we did not observe it in our own control samples. Based on our genetic results, *PRSS56* is a good candidate to be the causal gene for nanophthalmos in three of our four families. During the preparation of this manuscript, mutations in the predicted gene *PRSS56* were reported in patients with the related condition posterior microphthalmos [26,27], and in a related mouse ocular disorder [27].

As a relatively new gene, little is known about *PRSS56*. Its exonic structure as defined by RefSeq is provisional. The human exonic structure is based on a virtual assembly of multiple incompletely spliced cDNA clones, plus newly generated mouse full length cDNA clones. The gene is not yet annotated in the mammalian gene collection (MGC), ORFeome, or Vega (Vega genome blast) catalogs (except as a Vega possible pseudogene), and the Ensembl (Ensemble gene browser) structure prediction is incomplete compared to that in RefSeq. We have independently validated the predicted exon structure in mouse with a new cDNA clone obtained from embryonic mouse RNA. Because we used gene-specific primers to amplify first-strand total cDNA, the complete mRNA structure cannot be inferred from our sequence, only the likely open reading frame. We were unable to amplify or clone a full length cDNA from commercial human fetal brain RNA; either due to degradation of the sample or low expression level. *PRSS56* is expressed broadly at low levels, slightly higher in a variety of tissues according to the GeneCards database of GNF data, including testis, lymph node, brain, retina, and smooth muscle, though only possibly in pineal gland in the more current BioGPS. Its appearance in brain and retina seems most relevant to its proposed role in regulation of eye development. Bioinformatic analysis predicts a serine protease functional activity for *PRSS56*, however this remains to be demonstrated biochemically.

## 

**Table:** The additive used for Exon3/4 was 1,2-propanediol, 0.6 μl per 10 μl PCR reaction, to counteract high GC content. All reactions were at 60 °C except exon 3/4 which was at 55 °C.
